# Indigenous Knowledge and Traditional Uses of *Vangueria infausta* subsp. *infausta* Burch in Northern KwaZulu-Natal, South Africa

**DOI:** 10.3390/plants14121820

**Published:** 2025-06-13

**Authors:** Samukelisiwe Clerance Ngubane, Zoliswa Mbhele, Nontuthuko Rosemary Ntuli

**Affiliations:** Department of Botany, Faculty of Science, Agriculture and Engineering, University of Zululand, Richards Bay 3886, South Africa; ngubanes@unizulu.ac.za (S.C.N.); ntulir@unizulu.ac.za (N.R.N.)

**Keywords:** *Vangueria infausta* Burch, top priority fruit tree, food security, underutilized, ethnobotany, wild medlar

## Abstract

Indigenous top-priority fruit trees, like *Vangueria infausta* subsp. *infausta.* Burch (wild medlar), are essential for food security, climate resilience, and biodiversity. However, they remain underutilized due to limited documentation and integration into agricultural systems. This study presents the first ethnobotanical assessment of the wild medlar in Oyemeni, northeastern KwaZulu-Natal, South Africa. Surveys and interviews were conducted with 100 rural participants to explore its traditional uses, commercialization potential, and knowledge transmission. The findings reveal that the fruit is widely used in porridges, juices, and traditional beer, offering nutritional benefits such as vitamin C. Medicinally, it is valued for promoting oral health, wound healing, and spiritual protection. However, traditional knowledge is declining, particularly among the youth, due to generational shifts and modernization. The study highlights sustainable commercialization opportunities, such as value-added products and agroforestry integration, while emphasizing the need to preserve indigenous knowledge. These findings contribute to food security, biodiversity conservation, and cultural preservation in a changing socio-economic landscape. Future research should focus on biochemical evaluation of the plant’s medicinal properties and cross-regional comparisons.

## 1. Introduction

**Figure 1:**
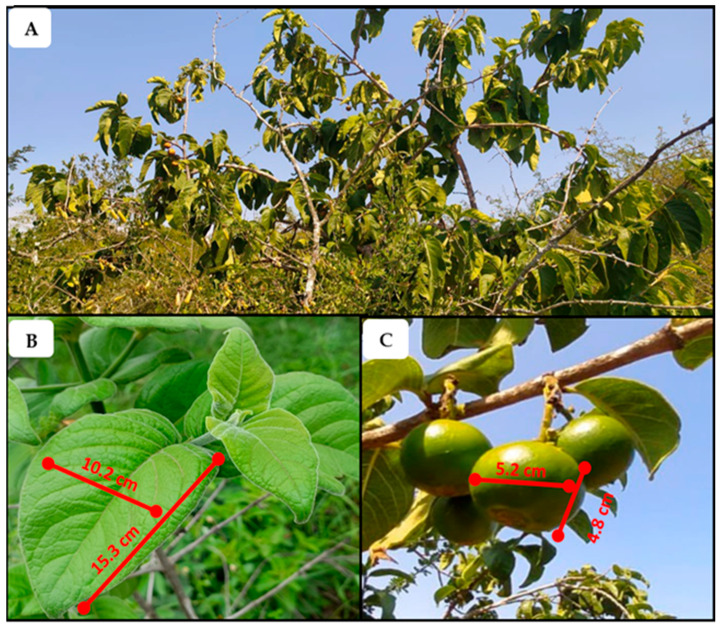
Images of *Vangueria infausta* subsp. *Infausta* wild medlar plant: (**A**)—tree; (**B**)—leaves; (**C**)—fruits (unripe).

Indigenous fruit trees play a critical role in food security, climate resilience, and biodiversity conservation, yet many remain underutilized and poorly documented, limiting their integration into sustainable agriculture [1]. Despite their ability to withstand climate stressors and their rich nutritional profiles, these species are largely excluded from mainstream agriculture, reducing their contribution to sustainable food production [2]. One such neglected species is *Vangueria infausta* subsp. *infausta* Burch (Figure 1), commonly known as wild medlar (English) or *umViyo* (isiZulu), a drought-tolerant fruit tree, native to southern Africa, with a long history of traditional use in food, medicine, and household applications [3,4]. However, its role in contemporary food systems, its commercialization potential, and the transmission of indigenous knowledge related to its use remain critically understudied.

The loss of indigenous knowledge exacerbates the marginalization of species like wild medlar, which is identified as one of the top priority fruit trees with economic potential in KwaZulu-Natal, South Africa [5]. Modernization, urbanization, and language loss have contributed to the decline of traditional food practices, threatening local food security [6]. In South Africa, where child malnutrition and food insecurity persist, underutilized indigenous fruits provide an opportunity to enhance local diets with nutritionally rich, climate-resilient alternatives to commercial crops [7]. However, because knowledge about these species is primarily transmitted through oral traditions, it is at risk of being lost as younger generations shift away from traditional food practices [8]. Without systematic documentation and formal integration into food security strategies, these valuable species remain underutilized, and their full potential remains unrealized [9].

Beyond its nutritional and cultural significance, the wild medlar also holds promise for climate adaptation and agricultural diversification. Yet, despite its ecological resilience and socio-economic value, it remains largely overlooked in policy frameworks. Existing studies have explored its food applications in Mozambique [10] and Zimbabwe [11], but its utilization in South Africa has received little attention. Addressing this research gap is particularly urgent in the wake of the COVID-19 pandemic, which exposed vulnerabilities in global food supply chains while underscoring the resilience of Indigenous Peoples’ biocultural food systems [12].

This study provides the first systematic ethnobotanical assessment of wild medlar in South Africa, documenting its traditional uses, processing methods, and knowledge transmission dynamics. By capturing and analyzing local knowledge, this research bridges the gap between traditional practices and sustainable commercialization, offering insights for conservation, climate adaptation, and food security policies. We provide empirical data on the species role as a resilient food source in biodiversity conservation, and sustainable agriculture, laying the foundation for policy integration and broader use as a valuable economical food.

## 2. Results

### 2.1. Socio-Demographic Information

**Figure 2:**
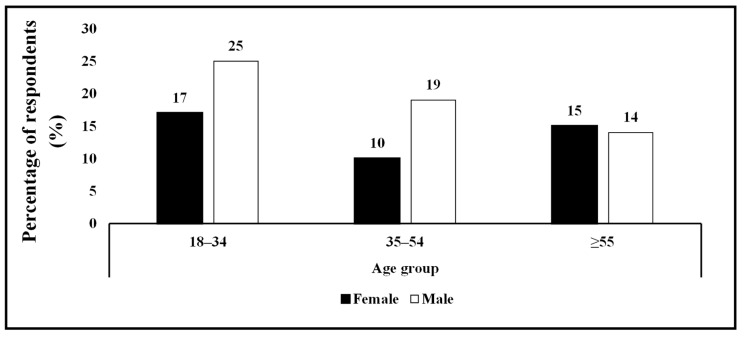
Percentage participants of Oyemeni locals by age groups.

A total of 100 Oyemeni locals were interviewed to document their knowledge on the uses and morphological variation in the wild medlar. More males (58%) participated compared to females (42%) (Figure 2). This pattern also persisted in the 18–34 and 35–54 age groups, where more males were represented. However, in the age group ≥ 55, there were more female participants than male.

### 2.2. The Significance of Umviyo, the Isizulu Name of Vangueria infausta (Wild Medlar)

In the Oyemeni community, all participants were familiar with the isiZulu name “*umViyo*”, which translates to a “group of warriors”. However, there were varying interpretations and connotations associated with the origin of this name. It was suggested that the name *umViyo* is derived from the resemblance of ripe brown fruits of the wild medlar to the heads of warriors hidden in the bushes during times of war. This visual similarity led to the name being associated with bravery and strength. Additionally, there is a belief that when wild medlar plants grow within or around homesteads, it is a strategic move by the ancestors to protect against war attacks and ward off negative energies. Therefore, these plants are considered sacred and are traditionally not removed due to their perceived protective value.

### 2.3. Consumption of Vangueria infausta (Wild Medlar) Fruit by the Community of Oyemeni

**Figure 3:**
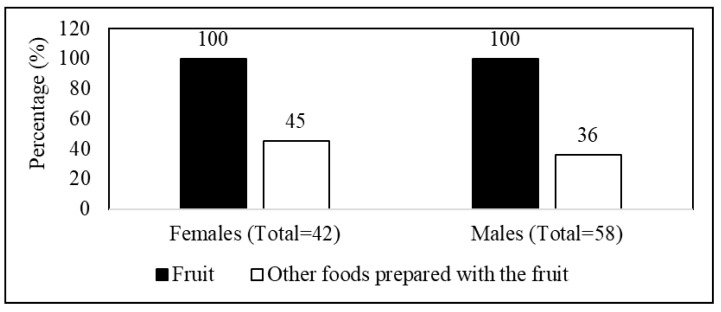
An overview percentage report on *Vangueria infausta* subsp. *infausta* (wild medlar) food uses by males and females of Oyemeni.

All participants were familiar with the fruit of the wild medlar. This is reflected by 100 participants (making up 100%) mentioning the fruit in the food category of Figure 2. The participants further shared various food preparations incorporating the wild medlar fruit into indigenous dishes (Table 1). However, the processing of the fruit into other foods was not as common as its direct fruit consumption. Females were more knowledgeable (by 45% of total females) about the other food uses of the fruit than males (with 36% of total males), despite having more male participants in the study (Figure 3 and Table 1). The people in Oyemeni presented various ways that the fruit of the wild medlar is utilized.

Direct fruit consumption was familiar and practiced by all (100%) participants, constituting of 58 males and 42 females (Table 1). The highest number of male participants (25) belong to the 18–34 age group, and the lowest (14) belong in the ≥55 age group. The highest response (17) for the female group was also recorded in the 18–34 age group, and the lowest response (10) was in the 35–54 age group.

A total of 13 (13%) participants reported the preparation of *umBhantshi*, a fruit-flavoured maize meal, as one of the dishes they make with wild medlar fruit (Table 1). Among them, six were males and seven were females. This dish was the second most frequently mentioned after direct fruit consumption. To prepare the fruity maize meal, the crushed fruits of the wild medlar are mixed with water using hands. The seeds and skin are removed from the mixture. The resulting puree is poured into a dish with crumbly maize porridge (*uPhuthu*) or crushed preboiled maize (*umCaba*) and mixed well before serving. Sugar is sometimes added for flavour.

Nine percent of participants in this study utilized the ripe wild medlar fruit as a flavouring for soft maize porridge (Table 1). This was reported by four males and five females. The process of making soft maize porridge involves crushing wild medlar fruits to create a fruit paste, which is then mixed in a pot with boiling water. In a separate bowl, a mixture of maize meal and water is prepared and subsequently added to the boiling fruit mixture. This mixture is stirred until smooth and allowed to simmer. The porridge can be served with or without sugar. Alternatively, fruit pulp can be added to a standard cooked porridge.

*Vangueria infausta* subsp. *infausta* in the Oyemeni area, 6% of the participants (Table 1) reported that juice from this fruit can be made by squeezing the pulp into water and mixing the contents. The seeds and skin are then removed, and sugar is added to taste. According to 5% of the participants, wild medlar fruits play a crucial role in this community by enhancing the flavour of alcohol, particularly the traditional beer (Table 1). They are commonly utilized when traditional beer lacks the desired level of acidity. In such cases, the fruit pulp is mixed with a small amount of water and added to the fermenting beer.

Fermented maize porridge (*amaHewu*) was mentioned by three participants as a composite of wild medlar fruit (Table 1). Like the alcohol production explained earlier, the wild medlar fruit pulp is added to fermenting maize porridge to enhance the flavour and increase the sour taste of the fermenting porridge. Two participants reported wild medlar fruit as a perfect addition to a fruit salad. The sweet–sour taste of wild medlar pulp was noted as a favourable addition to fruit salad and other sour desserts. To prepare a fruit salad, wild medlar fruits are cut, deseeded, and mixed with other fruits. In a dessert, the fruit pulp can be mixed in or used as a topping. According to one participant, an unripe wild medlar fruit is added to milk to induce milk curdling, a quick method of making *Amasi*.

One participant mentioned that wild medlar fruits are used to enhance a sour flavour in a sorghum porridge (*umNcindo*) (Table 1). A sour sorghum porridge is a meal made with crushed germinated sorghum seeds, maize meal, and wild medlar fruit pulp. These ingredients are mixed in warm water and left to ferment for 1–2 days. After fermenting, the mixture is cooked, to make a sour soft porridge. Apparently, this porridge is commonly used to produce traditional beer, where fruit addition enhances beer taste.

### 2.4. Medicinal and Other Uses of Vangueria infausta (Wild Medlar)

A total of 21 participants mentioned that the wild medlar plays a vital role in maintaining good oral health within the community (Table 2). The most common non-food uses of the wild medlar in the Oyemeni area were tooth brushing (11%) and treatment of toothache (10%). The leaves of this plant are utilized for brushing teeth by carefully wiping away dirt from the teeth using fresh velvety leaves. Afterward, water is swirled in the mouth and then spat out. Additionally, for treating toothache, wild medlar leaves are crushed and applied as a paste to the affected tooth. Alternatively, the roots are chopped, boiled, and cooled, and the mixture is used as mouth wash.

According to eight participants, the wild medlar tree is believed to possess protective powers against evil spirits in the homestead (Table 2). It is believed that elders would intentionally plant this tree within or around the homestead, such as in the livestock kraal or on either side of the household entrance. The purpose of this strategic plantation is to provide protection against evil spirits and shadows of dark “omen”. According to four participants, the leaves and roots of the wild medlar are used to create a lucky charm known as *iNtelezi* for warriors. A concoction is prepared from the plant and sprayed onto warriors before they engage in warfare or participate in traditional ceremonies involving stick fights. The belief is that the protective powers of the plant can shield the warriors from harm and promote unity among them.

Five participants mentioned that the wild medlar tree should not be used as firewood (Table 2). Although they were unsure of the reasons, it was noted that this information is instilled at a young age, where boys are taught to avoid using the plant in cattle fields, and girls are advised not to collect and bring the wood home. On the contrary, 4% mentioned that the plant makes good firewood. Five participants mentioned a traditional practice (for many years) to stop bedwetting, where the child climbs up the wild medlar tree and urinates from its top. It is believed that the natural powers possessed by the tree would aid the child to overcome bedwetting. A child can also lay the leaves under the sleeping mat or sheets for the same purpose. According to four participants, the leaves of the wild medlar were identified as a natural alternative to toilet paper for personal hygiene purposes. The selection of these leaves was based on their texture and width, with the most preferred leaves being soft and wide.

Four participants mentioned that the leaves of the wild medlar are used to treat various skin conditions, infections, burns, and cuts (Table 2). The paste from crushed leaves is applied onto the affected area. Additionally, water can be added to the crushed leaves, and this mixture is used for bathing the entire body. Alternatively, the ash from burnt branches is mixed with petroleum jelly and applies to the wound. One participant mentioned that leaves of the wild medlar are used to cover a wounded area to prevent over bleeding.

According to three participants, the stem of the wild medlar is utilized to prepare a treatment for stomach-ache (Table 2). The chopped stems are boiled, cooled, and strained to create a medicinal solution administered as an enema. Two participants also mentioned that the wild medlar can be used to prevent miscarriages in cows with a history of miscarriages. A stick from the wild medlar plant is gently used to tap the back of a cow, or alternatively, a string of leaves is wrapped around the lower back of the cow. The leaves of the wild medlar were a useful resource for wiping off fat from dishes and traditional meat platters (*uGqoko*). In traditional practice, a meat platter should not be washed with water until its next use, and that is when the leaves of the wild medlar come in handy for cleaning.

According to one participant, the root of the wild medlar can be used to treat earache (Table 2). The chopped roots are boiled in water and cooled. Once the mixture reaches a lukewarm temperature, a few drops are transferred into the affected ear. It is left to interact for a while before being drained out. One participant reported that roots of the wild medlar are also added to boiling water for steaming purposes (Table 2). Steaming is believed to open body pores and allow the herbs to enter the body.

One participant reported that the roots of the wild medlar are used to cleanse the digestive system by voluntarily induced vomiting. The chopped roots are boiled, and a cup of the boiled concoction is diluted into two litres of water (Table 2). This mixture is consumed to fill up the stomach and induce vomiting. A voluntary induced vomiting is recommended in the morning on an empty stomach. A participant in Oyemeni mentioned that this as a regular practice for internal body cleansing.

## 3. Discussion

This study expands the understanding of the socio-cultural, and claimed nutritional, and medicinal significance of the wild medlar as a priority fruit tree, in the Oyemeni community. By documenting indigenous knowledge beliefs and practices, it provides valuable insights into the influence of gender, age, and modernization on knowledge transmission, as well as the socio-cultural and medicinal importance of the species. This study offers a novel perspective on how traditional food and medicinal practices persist in contemporary society and their implications for cultural heritage, nutrition, and community resilience. The findings present a strong case for the integration of indigenous knowledge into conservation strategies and public health interventions, highlighting the importance of preserving traditional ecological knowledge.

### 3.1. Socio-Demographic Information

A higher proportion of males (58%) than females (42%) were interviewed about wild medlar uses in the Oyemeni area (Figure 3). This pattern was consistent among participants aged 18–34 and 35–54 years but reversed in the ≥55 age group. The greater percentage of male participants suggests a gender gap in knowledge about wild medlar, likely due to their frequent exposure to forests and bushes while herding livestock, hunting, or foraging. In contrast, women predominantly engage in household chores, limiting their direct interaction with wild plant species [13]. These findings highlight the influence of socio-cultural roles on engagement with nature and indigenous knowledge transmission [14]. Understanding these gendered patterns is crucial for designing inclusive conservation and knowledge preservation strategies.

Interestingly, the ≥55 age group was the only group where more females reported traditional uses of wild medlar than males. However, this was only observed in the food uses as fruit (15%), fermented maize meal (46%), and juice (33%). This may be attributed to their limited exposure to modernization in their upbringing, such as in the form of electricity and technology, which necessitated a deeper reliance on traditional ecological knowledge for survival. The findings suggest that modernization has played a significant role in shaping knowledge sharing across generations. Gendered subsistence and socio-cultural patterns indicate that men typically spend more time outdoors and travel greater distances than women, further shaping their knowledge acquisition [13,15]. This highlights a critical gap in intergenerational knowledge transfer, where older women retain substantial ethnobotanical knowledge, but younger generations may not have the same exposure.

Furthermore, the perception of local women as vulnerable and underestimated has contributed to a widening intergenerational knowledge gap, adversely affecting communities [16]. Additionally, the irregular transmission of ethnobiological knowledge across communities is influenced by various factors, including gender, which plays a crucial role in how traditional knowledge is upheld [17]. In this study, females presented a lower percentage (42%) of willing participants despite the wild medlar being well known as a food resource, and females are traditionally known as food handlers. The similar pattern of knowledge distribution among the two genders was portrayed in other uses as well. As modernization progresses, traditional knowledge is at risk of being lost, with potential long-term consequences for cultural heritage and local survival strategies [18]. This underscores the importance of fostering gender-inclusive approaches in indigenous knowledge systems to ensure that valuable ecological knowledge is preserved and effectively shared.

The implications of these findings extend beyond knowledge transmission; they can contribute to community resilience and adaptive strategies in response to environmental changes [19]. Addressing gender gaps in knowledge dissemination can enhance sustainable resource management and cultural conservation efforts. Future research should explore targeted interventions that empower women in indigenous knowledge systems and examine how modernization can coexist with traditional practices [20]. Developing inclusive educational programmes that integrate traditional ecological knowledge into formal learning structures could encourage both men and women to participate in preserving and sharing indigenous knowledge beliefs [21]. Such initiatives could strengthen community resilience while ensuring that critical ethnobotanical knowledge is safeguarded for future generations.

### 3.2. Significance of “Umviyo”, the IsiZulu Name of Wild Medlar

All participants recognized the isiZulu name “*umViyo*,” meaning “group of warriors”, though interpretations of its origin varied. Some suggested that the name derives from the resemblance of the ripe brown fruits of wild medlar to the heads of warriors concealed in the bushes during battle, symbolizing bravery and strength. This recognition highlights the deep cultural significance embedded in plant nomenclature [22]. The varying interpretations suggest that indigenous knowledge is dynamic, shaped by historical narratives and lived experiences [23].

The name *umViyo* not only reflects the physical attributes of the plant but also embodies symbolic associations with resilience and protection. This aligns with the doctrine of signatures, a traditional concept where the appearance of a plant is believed to indicate its uses [24]. The association of wild medlar trees with protection extends beyond symbolism as many interviewees mentioned that planting it near homesteads serves as a safeguard, believed to offer ancestral protection against attacks in war and negative energies. This perception renders the plant sacred, reinforcing cultural taboos against its removal. Similarly to other isiZulu plant names, *umViyo* is derived from a combination of factors, including growth form, habitat, effects, appearance, and uses [25]. In Zulu culture, plant names often serve a mnemonic function, aiding diviners and traditional healers in recalling the various medicinal and spiritual functions of plants [26]. This function plays a crucial role in the oral transmission of indigenous knowledge, ensuring that essential ecological and cultural information is preserved across generations [27].

The integration of cultural beliefs into conservation strategies can enhance community engagement and foster sustainable environmental management. Similar approaches have been successfully applied in other indigenous communities, where traditional ecological knowledge has informed biodiversity conservation initiatives [28]. The findings of this study underscore the interconnectedness of natural and cultural systems within the Zulu context. Recognizing and respecting these cultural values is essential for developing inclusive conservation policies that preserve both biodiversity and indigenous heritage [29].

### 3.3. Consumption of Wild Medlar Fruit

Variation in the consumption of wild medlar fruits across different gender and age groups is presented in Table 1. All participants were familiar with the direct consumption of wild medlar fruits, with many sharing various methods of preparing them. The popularity of direct consumption in the Oyemeni community mirrors patterns observed in the Mapulana community of Mpumalanga province, where sun-dried fruits are enjoyed as snacks [30]. The taste of wild medlar fruit has been likened to a combination of green apple (*Malus pumila* Miller), medlar (*Mespilus germanica* L.), and pineapple (*Ananas comosus* (L.) Merr.) [3,31]. Furthermore, this fruit is rich in calcium, potassium, magnesium, sodium, phosphorus, iron, and zinc [32], and its complex flavour profile encourages its incorporation into other foods. Interestingly, evidence for the greatest consumption of the fruit came from the younger generation, suggesting a promising opportunity to preserve and document indigenous knowledge for future generations.

The study also revealed a significant gender-based difference in culinary knowledge. Despite a higher number of male participants (being 58% of total participants), when it came to additional culinary uses of the fruit, there were more participants in the female group (45%) who presented the information than in the male group (36%) (Figure 3). This finding emphasizes the crucial role women play in food preparation and the transmission of culinary knowledge within the community [33]. It also suggests that empowering women and recognizing their contributions to food culture could be beneficial in preserving these traditions [34], despite them representing a lower percentage of willing participants in this study.

One of the most popular dishes was *umBhantshi*, a fruit-flavoured maize meal prepared using wild medlar fruits (13 reports). To prepare this dish, the fruit is hand-crushed, its seeds and skin removed, and the resulting puree is combined with crumbly maize porridge (*uPhuthu*) or crushed preboiled maize (*umCaba*). The mixture may be sweetened with sugar before serving. Interestingly, the preparation of *umBhantshi* from wild medlar differs slightly from the version made with *Strychnos spinosa* (monkey orange), where the mixture is allowed to ferment before consumption [35]. Given that maize meal is a staple food in South Africa [36], the addition of wild medlar fruit enhances both the flavour and nutritional value of this common dish.

A smaller proportion of participants (9%) reported using ripe wild medlar fruits to flavour soft porridge, a practice that involves crushing the fruit into a paste and mixing it with boiling water, then adding maize or sorghum meal. This method is documented for the first time in this study. Other indigenous fruits, such as *Adansonia digitata* L. (baobab) and *Uapaca kirkiana* Mull. Arg. (wild loquat), *Parinari curatellifolia* Planch. Ex Benth. (mobola plum), *Strychnos cocculoides* Baker (corky-bark monkey orange), and *Xantocercis zambesiaca* (baker) Dumaz-le-Grand (nyala berry) have also been reported to enhance the flavour of maize or sorghum porridge [11,37].

Six percent of participants reported preparing wild medlar juice by squeezing the pulp into water, mixing it, and removing the seeds and skin, with sugar added for sweetness. Additionally, 5% of our sample mentioned using wild medlar fruits to enhance the flavour of traditional beer, particularly when the beer lacks the desired acidity. In these cases, the fruit pulp is mixed with a small amount of water and added to the fermenting beer. The participants mentioned that the wild medlar fruits added a unique tartness and depth to the beer, creating a more complex flavour profile.

Fermented maize porridge (*amaHewu*) was mentioned by three participants, where the fruit pulp is added to fermenting maize porridge to enhance its sourness and flavour. Similarly, one of the participants mentioned that wild medlar fruit is used to sour sorghum porridge (*umNcindo*), which is made from germinated sorghum seeds, maize meal, and wild medlar fruit pulp, then left to ferment for 1–2 days before cooking. This dish, often used in the production of traditional beer, demonstrates the versatility of wild medlar in enhancing the flavour of fermented foods and beverages. The use of fruit pulp could introduce beneficial specific microbes that alter the fermentation profile, potentially influencing the acidity, probiotic content, and texture of the porridge [38].

One of the interviewees also mentioned that unripe, chopped wild medlar fruits are added to fresh milk to induce curdling, a traditional method used to make Amasi, a fermented dairy product common in southern Africa [39]. This application of wild medlar in curdling milk is significant, as the search for alternative milk coagulants is growing in response to increasing dairy product demand [40]. The knowledge of wild medlar curdling ability contributes to food security research, offering an alternative solution for dairy processing in resource-limited areas.

The sour taste, low moisture content, and apple–pineapple flavour of wild medlar make it ideal for diversifying and enhancing various beverages in the Oyemeni area, including juices, alcoholic drinks, and fermented porridge. Outside of Oyemeni, wild medlar has also been reported to enhance the flavour of various beverages [3,41,42]. The addition of *V. infausta* to these beverages contributes to the daily caloric intake of community members, as sugars and organic acids in the fruit improve the overall taste [43].

Two participants also reported using wild medlar in fruit salads, where its sweet–sour taste complements other fruits and desserts. The fruit is deseeded, cut, and mixed with other fruits, or incorporated into desserts as a topping. The inclusion of indigenous fruits like wild medlar in fruit salads and desserts not only enhances flavour but also promotes nutritional diversity and provides probiotic benefits [44]. This practice could be encouraged for other indigenous fruits, helping to bridge traditional and modern culinary practices and promoting greater consumption of local fruits [45].

The recognition of wild medlar fruit for direct consumption and in diverse culinary preparations underscores its cultural significance in the Oyemeni community. This reinforces the connection between traditional food practices and cultural identity, showing how indigenous knowledge shapes dietary habits [46]. The higher response rates from the younger generation regarding the uses of wild medlar suggest a potential for revitalizing and preserving indigenous knowledge. This is also in agreement with the versatility hypothesis which predicts that people are more likely to preserve knowledge to plant that has a greater number of applications to human [23]. Therefore, documenting these culinary practices can ensure that this knowledge is passed down and remains relevant even in the younger generation, fostering a sense of identity and continuity within the community [47].

The nutritional richness of wild medlar fruits, including essential minerals like calcium, potassium, and magnesium, underscores their potential role in enhancing food availability. Incorporating these fruits into staple dishes like porridge and traditional beverages can improve dietary diversity and nutrition in the community [46]. The fruit’s unique flavour profile encourages culinary creativity, as its incorporation into various dishes such as porridge, juice, and traditional beer reflects the community’s adaptability in using local ingredients to enhance flavour and nutrition [48]. The detailed descriptions of traditional dishes, such as *umBhantshi*, and the use of wild medlar in fermentation processes indicate a rich culinary heritage that should be preserved and documented. These findings highlight the need to bridge traditional and modern culinary practices to enhance food security and preserve cultural heritage.

### 3.4. Medicinal and Other Uses of Wild Medlar

The medicinal and practical uses of wild medlar within the Oyemeni community are many and multifaceted, again reflecting the deep connection between the community and their natural environment (Table 2). The use of wild medlar for oral health, particularly in tooth brushing and toothache treatment, is among its interesting applications. The fresh, velvety leaves are employed to clean teeth, and the plant is also used to prepare a mouthwash for toothache from chopped, boiled and cooled roots. The use of plant leaves for tooth brushing and dental pain has previously been reported in Mozambique [4,49], but this study uniquely contributes by highlighting the dual function of the plant’s leaves, both for cleaning and healing purposes. This suggests that wild medlar has a holistic role to healthcare that integrates oral hygiene with broader cultural practices.

Another significant finding was the plant’s spiritual and protective uses, particularly in homesteads and for warriors. The belief that wild medlar offers protection against evil spirits and is used in creating lucky charms known as *iNtelezi* adds a layer of cultural richness. These practices, aligned with other reports on the use of plants for spiritual protection [4,50], demonstrate a deeper cultural belief in the plant’s mystical properties. This cultural use reflects a broader pattern where plants are employed for their spiritual significance, as seen in other African communities, including the Zulu people’s use of plants to foster unity [26].

The contradictory views on using wild medlar as firewood as some participants advising against it use due to potential health risks, such as testicular swelling [51], highlight the complexities within indigenous knowledge systems. This demonstrates the importance of understanding the cultural context and traditional practices when considering the utilization of natural resources. It also underscores the need for further research and dialogue to bridge the gap between traditional knowledge and modern scientific understanding [52].

An unexpected finding in this study was the use of wild medlar in traditional practices to treat bedwetting in children. Climbing the tree or using its leaves under the sleeping mat is believed to cure the condition. This aligns with reports from other regions, where plants are used in non-ingestive ways to treat conditions like bedwetting [53]. This practice reflects a culturally rooted approach to child health that differs from conventional medical treatments.

Wild medlar leaves are reported as effective for treating skin conditions, infections, and burns. Crushed leaves, when applied to wounds, reflect its potential antimicrobial properties [54]. The leaves’ ability to stop bleeding, as well as the use of a stem concoction to treat stomach aches, introduces novel therapeutic uses of the plant that have not been widely documented. The application of wild medlar for wound healing and treatment of stomach and ear ailments demonstrates the plant’s potential as a source of bioactive compounds [55]. While no direct evidence exists regarding its coagulating compounds, the plant’s success in treating bleeding and serving as a wound dressing could inspire further pharmacological investigations into its potential therapeutic properties [56,57]. These findings contribute to the growing body of knowledge beliefs on the medicinal properties of wild medlar, particularly regarding wound healing, gastrointestinal ailments, and earaches. Interdisciplinary studies, such as phytochemistry and pharmacology, are commended to validate and characterize the bioactive compounds responsible for the reported traditional uses. The plant is also valued locally for its edible fruit, which can be incorporated into traditional dishes. Further scientific investigations can elevate the medicinal and nutritional value of the plant, thereby contributing to local economic development and enhancing public health outcomes.

In livestock, the reported use of wild medlar is traditionally believed to support livestock reproductive health. The wild medlar adds to the list of other plants used by local farmers in South Africa to prevent miscarriage in livestock [58]. This emphasizes the potential for integrating traditional knowledge into modern animal husbandry practices to improve livestock health and productivity [59]. The plant’s versatility also extends to practical uses, such as serving as a tool to wipe off fat from traditional meat platters (*uGqoko*). This application adds to the plant’s reputation as a versatile material, further reflecting its role in daily life.

## 4. Materials and Methods

### 4.1. Study Area

**Figure 4:**
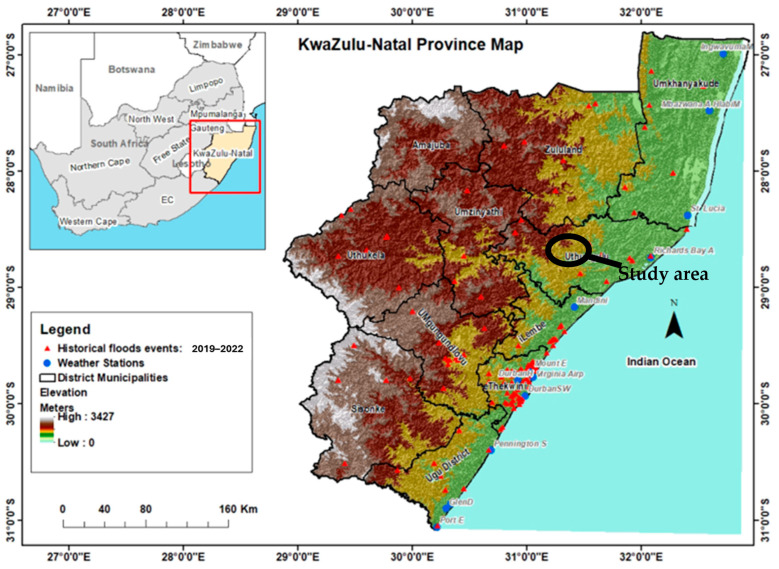
The geographical location of the study area, Oyemeni, is under the King Cetshwayo District Municipality (formerly known as Uthungulu) in KwaZulu-Natal, South Africa [62].

This study was conducted in Oyemeni (28°50′ S, 31°42′ E), a rural area within the Ongoye Reserve in northeastern KwaZulu-Natal, South Africa [33] (Figure 4). The region falls under the King Cetshwayo District Municipality, which has a population of nearly 203,000 with 80% residing in rural settlements. Socioeconomic data indicate that 86% of the population earns below $343 USD per month, with 41.3% earning between $43 USD and $171 USD [60]. Given these economic constraints, many residents depend on non-commercial food sources, including wild edible plants, for sustenance [61].

Oyemeni was selected for its high ethnobotanical relevance, particularly the prevalence of the wild medlar tree around homesteads and its widespread use of its fruits in traditional diets. The region’s rural communities retain deep-rooted indigenous knowledge related to food security and plant-based resource use [63]. This knowledge is critical in sustaining local livelihoods and enhancing resilience to food scarcity, particularly in marginalized communities such as Oyemeni [64]. A voucher specimen of *Vangueria infausta* subsp. *infausta* is at the University of Zululand herbarium (accession number UZULU962087).

### 4.2. Ethnobotanical Survey

To ensure ethical integrity and cultural sensitivity, formal approval was obtained from the local tribal court before engaging with community members. Data collection was conducted through structured, open-ended questionnaires, designed to document indigenous knowledge regarding the use of the wild medlar.

A stratified random sampling method was employed to ensure representative participation across different demographic groups. A total of 100 participants were selected from randomly chosen homesteads, with at least one knowledgeable and consenting adult interviewed per household. All participants were fully briefed about the study’s objectives in isiZulu, their native language, and provided informed written consent prior to participation. Ethical guidelines for ethnobotanical research, including confidentiality and respect for indigenous knowledge, were strictly adhered to throughout the study.

### 4.3. Questionnaire Design

The questionnaire consisted of three sections: socio-demographic data, which included participant gender, age group, and village of residence; local nomenclature and significance, which explored the etymology and cultural importance of *V. infausta*; and traditional uses, which documented the consumption of *V. infausta* fruit and the applications of other plant parts in food and other domains.

To ensure linguistic accuracy, the questionnaire was initially compiled in English and then translated into isiZulu. A back-translation process was conducted to verify consistency and cultural appropriateness. The questionnaire was also pre-tested with a small subset of 10 participants (*n* = 10) to assess their clarity of understanding and cultural relevance. Feedback from the pilot phase was incorporated to refine ambiguous questions. Open-ended prompts allowed participants to elaborate on their responses beyond predefined categories, ensuring the researchers a nuanced understanding of indigenous knowledge.

### 4.4. Data Analysis

A frequency index for variables given by the 100 participants was analyzed using the following formula that was adapted from [65]:

$$FI=\frac{FC}{N}\times 100,$$

where *FI* represents the frequency index, which denotes the percentage of frequency of the use of *V. infausta* by participants, *FC* represents the number of participants who mentioned a use of *V. infausta*, and *N* is the total number of participants. Within these variables, the frequency index was further calculated per gender, and age groups within each gender, where *FC* was the number of participants per age group, and *N*, the number of participants per gender [35]. Thus, the level of knowledge the percentage of a specific gender would be indicated per variable.

## 5. Conclusions

This study has demonstrated that the wild medlar serves as a culturally and nutritionally significant species within the Oyemeni community. Its various applications in food, medicine, and spiritual practices underscore the rich indigenous knowledge associated with this plant. However, while this study has inherent limitations in terms of generalizability due to its focus on a specific community. It has established novel baseline data for further research that can explore cross-regional comparisons using deeper qualitative and quantitative methods. The findings also provide a foundation for future research aimed at integrating traditional plant knowledge with modern conservation and healthcare strategies. Future research should also focus on the biochemical analysis of the wild medlar to evaluate its medicinal properties scientifically. Additionally, exploring potential commercialization avenues for the fruit and its products may create economic opportunities for rural communities. Furthermore, incorporating indigenous knowledge into school curricula can further strengthen knowledge transmission and contribute to the preservation of traditional practices in the long term.

## Figures and Tables

**Table 1:** Knowledge prevalence on indigenous foods prepared with *Vangueria infausta* subsp. *infausta* (wild medlar) in the Oyemeni area in terms of gender and age differences.

Food	Participants	Gender	Age Group (Years)		
		G [N (%)]	18–34 N (TP; TGP)	35–54 N (TP; TGP)	≥55 (TP; TGP)
Fruit	100	F [42 (42)] M [58 (58)]	17 (17; 40) 25 (25; 43)	10 (10; 24) 19 (19; 33)	15 (15; 36) 14 (14; 24)
Fermented maize meal (*umBhantshi*)	13	F [7 (54)] M [6 (46)]	0 (0; 0) 3 (23; 50)	1 (8; 14) 1 (8; 17)	6 (46; 86) 2 (15; 33)
Maize porridge	9	F [5 (55)] M [4 (44)]	5 (55; 100) 3 (33; 75)	0 (0; 0) 1 (11; 25)	0 (0; 0) 0 (0; 0)
Juice	6	F [2 (33)] M [4 (67)]	0 (0; 0) 2 (33; 50)	0 (0; 0) 1 (17; 25)	2 (33; 100) 1 (17; 25)
Alcohol	5	F [1 (20)] M [4 (80)]	1 (20; 100) 4 (80; 100)	0 (0; 0) 0 (0; 0)	0 (0; 0) 0 (0; 0)
Fermented maize porridge (*amaHewu*)	3	F [2 (67)] M [1 (33)]	0 (0; 0) 1 (33; 100)	2 (67; 100) 0 (0; 0)	0 (0; 0) 0 (0; 0)
Fruit salad	2	F [2 (100)] M [0 (0)]	1 (50; 50) 0 (0; 0)	1 (50; 50) 0 (0; 0)	0 (0; 0) 0 (0; 0)
Milk curdling (*Amasi*)	1	F [0 (0)] M [1 (100)]	0 (0; 0) 0 (0; 0)	0 (0; 0) 1 (100; 100)	0 (0; 0) 0 (0; 0)
Sour porridge (*umNcindo*)	1	F [0 (0)] M [1 (100)]	0 (0; 0) 1 (100; 100)	0 (0; 0) 0 (0; 0)	0 (0; 0) 0 (0; 0)

G—gender; F—female; M—male; N—number of gender participants; TP—total percentage; TGP—total gender percentage; %—frequency index.

**Table 2:** The frequency index of indigenous knowledge on medicinal and other uses of *Vangueria infausta* subsp. *infausta* (wild medlar) in the Oyemeni area across gender, total percentage within age group, and total percentage per gender within age groups (continued).

Use	Participants	Gender	Age (Years)		
		G [N (%)]	18–34 N (TP; TGP)	35–54 N (TP; TGP)	≥55 N (TP; TGP)
Toothbrushing	11	F [2 (18)] M [9 (82)]	1 (9; 50) 3 (27; 33)	1 (9; 50) 4 (36; 44)	0 (0; 0) 2 (18; 22)
Toothache	10	F [4 (40)] M [6 (60)]	4 (40; 100) 3 (30; 50)	0 (0; 0) 3 (30; 50)	0 (0; 0) 0 (0; 0)
Protection against evil spirits	8	F [0 (0)] M [8 (100)]	0 (0; 0) 2 (25; 25)	0 (0; 0) 3 (38; 38)	0 (0; 0) 3 (38; 38)
Not as firewood	5	F [1 (20)] M [4 (80)]	0 (0; 0) 0 (0; 0)	0 (0; 0) 3 (60; 75)	1 (20; 100) 1 (20; 25)
Wetting the bed	5	F [2 (40)] M [3 (60)]	1 (20; 50) 2 (40; 67)	1 (20; 50) 1 (20; 33)	0 (0; 0) 0 (0; 0)
As a toilet wipe	4	F [1 (25)] M [3 (75)]	0 (0; 0) 0 (0; 0)	0 (0; 0) 3 (75; 100)	1 (25; 100) 0 (0; 0)
Firewood	4	F [1 (25)] M [3 (75)]	0 (0; 0) 1 (25; 33)	0 (0; 0) 2 (50; 67)	1 (25; 100) 0 (0; 0)
Bring unity and courage for warriors	4	F [0 (0)] M [4 (100)]	0 (0; 0) 3 (75; 75)	0 (0; 0) 1 (25; 25)	0 (0; 0) 0 (0; 0)
Wound healing	4	F [3 (75)] M [1 (25)]	2 (50; 67) 1 (25; 100)	1 (25; 33) 0 (0; 0)	0 (0; 0) 0 (0; 0)
Stomachache	3	F [2 (67)] M [1 (33)]	1 (33; 50) 1 (33; 100)	1 (33; 50) 0 (0; 0)	0 (0; 0) 0 (0; 0)
Prevents cow miscarriage	2	F [1 (50)] M [1 (50)]	0 (0; 0) 0 (0; 0)	0 (0; 0) 1 (50; 100)	1 (50; 100) 0 (0; 0)
Wiping fat off the dishes	1	F [1 (100)] M [0 (0; 0)]	0 (0; 0) 0 (0; 0)	1 (100; 100) 0 (0; 0)	0 (0; 0) 0 (0; 0)
Stops bleeding	1	F [1 (100)] M [0 (0)]	0 (0; 0) 0 (0; 0)	1 (100; 100) 0 (0; 0)	0 (0; 0) 0 (0; 0)
Earache	1	F [0 (0)] M [1 (100)]	0 (0; 0) 1 (100; 100)	0 (0; 0) 0 (0; 0)	0 (0; 0) 0 (0; 0)
Steaming	1	F [1 (100)] M [0 (0)]	1 (100; 100) 0 (0; 0)	0 (0; 0) 0 (0; 0)	0 (0; 0) 0 (0; 0)
Vomiting	1	F [0 (0)] M [1 (100)]	0 (0; 0) 0 (0; 0)	0 (0; 0) 1 (100; 100)	0 (0; 0) 0 (0; 0)

G—gender; F—female; M—male; N—number of gender participants; TP—total percentage; TGP—total gender percentage; %—frequency index.

## Data Availability

The data presented in this study are available on request from the corresponding authors.
